# Novel Validated Stability-Indicating UPLC Method for the Estimation of Naproxen and its Impurities in Bulk Drugs and Pharmaceutical Dosage Form

**DOI:** 10.3797/scipharm.1207-12

**Published:** 2012-09-09

**Authors:** Papanaboina Venkatarao, Morrisetty Nagendra Kumar, Maram Ravi Kumar

**Affiliations:** 1 Dr. Reddy’s Laboratories Ltd. IPDO, Bachupally, Hyderabad-500072, A.P, India.; 2 Department of Chemistry, J. N. T. University, Kukatpally, Hyderabad-500072, A.P, India.

**Keywords:** Naproxen, Validation, UPLC, Stability-indicating, Degradation

## Abstract

A novel, reversed-phase ultra-performance liquid chromatographic method was developed and validated for the determination of related substances in Naproxen (NAP) bulk drugs and dosage forms. The related substances included degradation and process-related impurities. The method was developed using the Waters Acquity BEH C18 column using the gradient program with mobile phase A of a pH 7.0 phosphate buffer and methanol in the ratio of 90: 10 (v/v) and mobile phase B as methanol and acetonitrile in the ratio of 50:50 (v/v). Naproxen and its impurities were monitored at 260 nm. Naproxen was subjected to the stress conditions of oxidative, acid, base, hydrolytic, thermal, humidity, and photolytic degradations. The degradation products were well-resolved from the main peak and its impurities, proving the stability-indicating power of the method. The performance of the method was validated according to the present ICH guidelines for specificity, limit of detection, limit of quantification, linearity, accuracy, precision, ruggedness, and robustness.

## Introduction

Naproxen is (αS)-6-methoxy-α-methyl-2-naphthaleneacetic acid. Its empirical formula is C_14_H_14_o_3_ with a molecular weight of 230.26. Naproxen is a member of the aryl acetic acid group of nonsteroidal anti-inflammatory drugs used for the treatment of pain or inflammation caused by conditions such as arthritis, ankylosing spondylitis, or menstrual cramps [1]

The European Pharmacopoeia (EP) and United States Pharmacopoeia (USP) monograph methods for Naproxen-related compounds is by the thin layer chromatographic technique, which cannot separate all of the potential impurities and degradation compounds of Naproxen. Several liquid chromatography methods were reported for the individual and simultaneous determination of Naproxen and other anti-inflammatory compounds in human serum [2–5] and in pharmaceutical preparations [6–8]. One article is available for the estimation of Naproxen and its related compounds by HPLC using a porous graphitic carbon column [9], which did not discuss the degradation of Naproxen, and the reported method was with traditional HPLC. The major objective of this article is to develop a stability-indicating method to estimate Naproxen and its related compounds using a sub-2-μm particle column with UPLC. The usage of sub-2μm particles increases the efficiency and sensitivity, and decreases the retention volume when compared to conventional HPLC. It seems that in the future, UPLC systems with elevated pressure will replace the conventional HPLC gradually in all areas of liquid chromatography including pharmaceutical analysis [10]. According to our research, there is no stability-indicating UPLC method reported in the literature yet that can conduct an accurate and quantifiable analysis of Naproxen, related compounds of Naproxen in API, and the dosage forms.

**Fig. 1.**
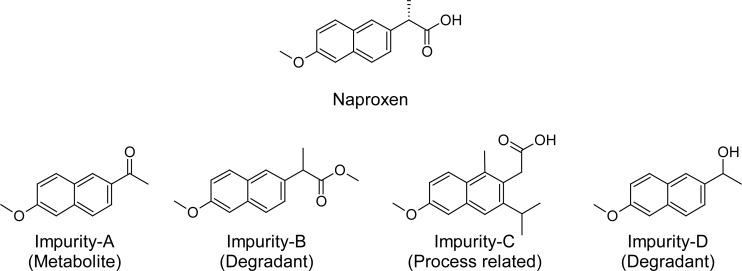
Chemical structures of Naproxen and its four impurities

Naproxen has four impurities named, Imp-A (metabolite), Imp-B (degradant), Imp-C (process related), and Imp-D (degradant) (Fig. 1).

## Materials and methods

### Chemicals and reagents

The Naproxen standard, the bulk drug, and the impurity standards with a purity of 99.8% were supplied by Dr. Reddy’s laboratories Ltd, Hyderabad, India. Commercially available Naprosyn 250mg tablets were used for the dosage form analysis. The HPLC grade methanol, acetonitrile, analytical grade potassium di hydrogen phosphate, orthophosphoric acid, triethylamine, borax, edetate disodium, and sodium hydroxide were purchased from Merck, Darmstadt, Germany. HPLC grade water was prepared using the Millipore Milli-Q Plus water purification system, Bedford, MA, USA.

### Chromatographic conditions and equipment

The LC system of Waters Acquity UPLC with a photodiode array detector was used for this study and chromatographic separation was achieved on the Acquity BEH-C18 (50 mm × 4.6 mm × 1.7μm) column as the stationary phase. The separation was achieved by the gradient method. Mobile phase A contained a mixture of 8.0mL of triethylamine (TEA) in a 20mM KH_2_PO_4_ buffer (adjusted to a pH of 7.0 with orthophosphoric acid) and methanol in the ratio of 90:10 (v/v/v), respectively. Mobile phase B contained a mixture of methanol and acetonitrile in a ratio of 50:50 (v/v), respectively.

The flow rate of the mobile phase was 0.3 mL/min. The UPLC gradient program (T/%B) was set as 0.01/20, 2.0/30, 5/50, 6.0/70, 8.5/70, 9.5/20, and 11/20. The column temperature was maintained at 40°C and the injection volume was 3.0 μL. The peaks were monitored at the wavelength of 260 nm.

### Preparation of diluent, standard, and sample solution

The diluent used for the standard and sample preparation was a mixture of methanol and water in the ratio of 80:20 (v/v).

A stock solution of NAP 2500 μg/mL was prepared by dissolving an appropriate amount of the drug in the diluent. Working solutions containing 2.5 μg/mL and 250 μg/mL were prepared from the stock solution and used for the determination of related substances and for the assay determination, respectively. Individual impurity stock solutions were prepared, diluted, and mixed to get 2.5 μg/mL, which were then used for the method validation of NAP.

At least 20 tablets of Naproxen were weighed and crushed to a fine powder by a mortar and pestle. An accurately weighed amount of the above powder equivalent to 250 mg of Naproxen was added to a 100 mL volumetric flask, with about 70 mL of diluents added, which was then sonicated for 20 minutes with intermediate shaking, and made up to the volume with diluent. The above solution was then filtered through a 0.22 μm Nylon membrane filter and injected into the UPLC. An aliquot of 1.0mL of this solution was diluted to 10 mL with the buffer solution, yielding 250μg/mL of solution that was filtered through a 0.22 μm Nylon membrane filter and injected into the UPLC.

### Method validation

The proposed method was validated as per ICH guidelines [11, 12].

#### Specificity

Specificity is the ability of the method to measure the analyte response in the presence of its potential impurities. To prove the stability-indicating nature of the method, NAP samples were exposed to acid, base, peroxide, heat, water, humidity, and photolytic stress conditions, and then injected into the UPLC. Peak purity was checked for NAP peaks by using the PDA detector in the stress samples. The purity angle was within the purity threshold limit obtained in all the stressed samples and demonstrated the analyte peak homogeneity.

The assay of stressed samples was performed by comparison to the reference standard, and the mass balances (% assay + % impurities + % degradation products) were calculated.

#### Precision

The precision of the related substances method was verified by repeatability and by intermediate precision. Repeatability was checked by injecting six individual preparations of the NAP real sample spiked with 0.10 % of its four impurities. % RSD of the area for each impurity was calculated, and the intermediate precision of the method was also evaluated using different analysts and performing the analysis on different days. Precision of the assay method was evaluated by carrying out six independent assays of real samples of NAP at the 250 μg/ml level against the qualified reference standard. The intermediate precision of the assay method was evaluated by different analysts.

#### Limit of Detection (LOD) and Quantification (LOQ)

The LOD and LOQ for impurities were determined at a signal–to-noise ratio of 3:1 and 10:1 respectively, by injecting a series of dilute solutions with known concentrations. The precision study was also carried out at the LOQ level by injecting six individual preparations of impurities and calculating the % RSD of the area.

#### Linearity

Linearity test solutions for the related substances method were prepared by diluting the impurity stock solution to the required concentrations. The solutions were prepared at six concentration levels from the LOQ to 200 % of the specification level (LOQ, 0.025, 0.1, 0.125, 0.15, and 0.20 %).

Linearity test solutions for the assay method were prepared from the Naproxen stock solution at six concentration levels from 50 to 150% of the assay analyte concentration. The peak area versus concentration data was treated by least-squares regression analysis.

#### Accuracy

The accuracy study was carried out in triplicate using four concentration levels (LOQ, 50 %, 100 %, and 150 %). Standard addition and recovery experiments were conducted on the real sample to determine the accuracy of the related substances method. The accuracy of the assay method was evaluated in triplicate using three concentration levels (50 % to 150 %) in the real sample.

#### Robustness

To determine the robustness of the developed method, experimental conditions were deliberately changed and the tailing factors for Naproxen and its impurities were recorded. The flow rate of the mobile phase was 0.3 ml/min to study the effect of flow rate on the resolution; flow was changed by 0.03 units from 0.27 to 0.33 ml/min. The effect of the column temperature on the resolution was studied at 20°C and 30°C instead of at 25°C.

#### Solution stability

The solution stability of Naproxen in the assay method was carried out by leaving both of the test solutions of the sample and reference standard in tightly capped volumetric flasks at room temperature for 24 h. The same sample solutions were assayed for 12-h intervals up to the study period. The solution stability of Naproxen and its impurities in the related substance method was carried out by leaving spiked sample solutions in tightly capped volumetric flasks at room temperature for 24h. The contents of impurities 1–4 were determined for every 12-h Interval up to the study period.

## Results and Discussion

### Method Development and Optimization

**Fig. 2.**
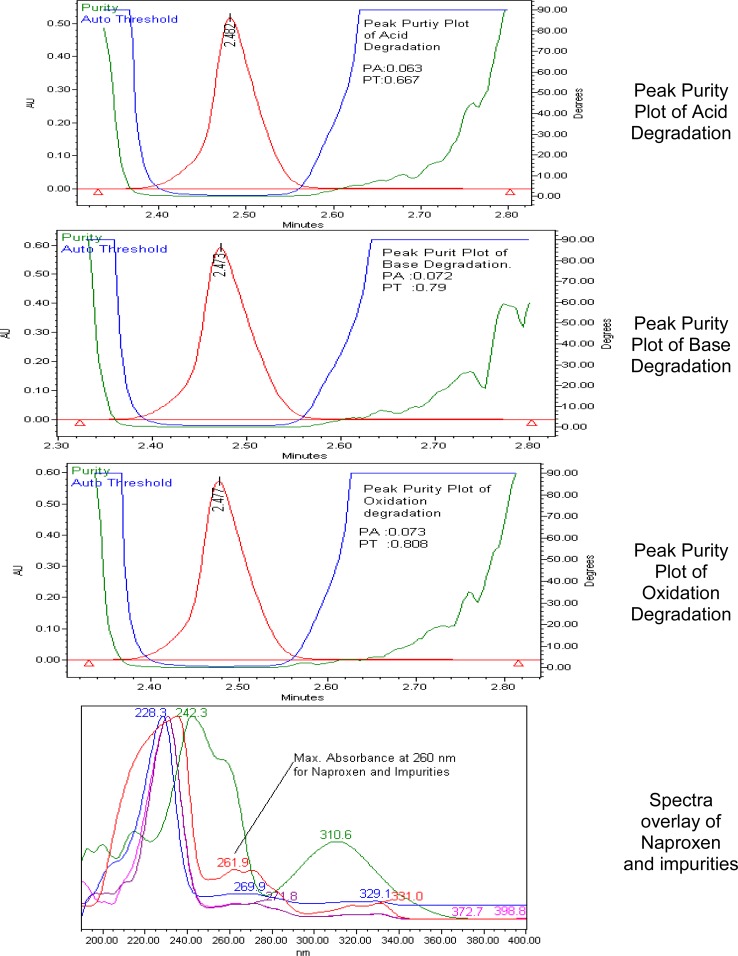
Peak purity plots for stress studies and spectra overlay

**Fig. 3.**
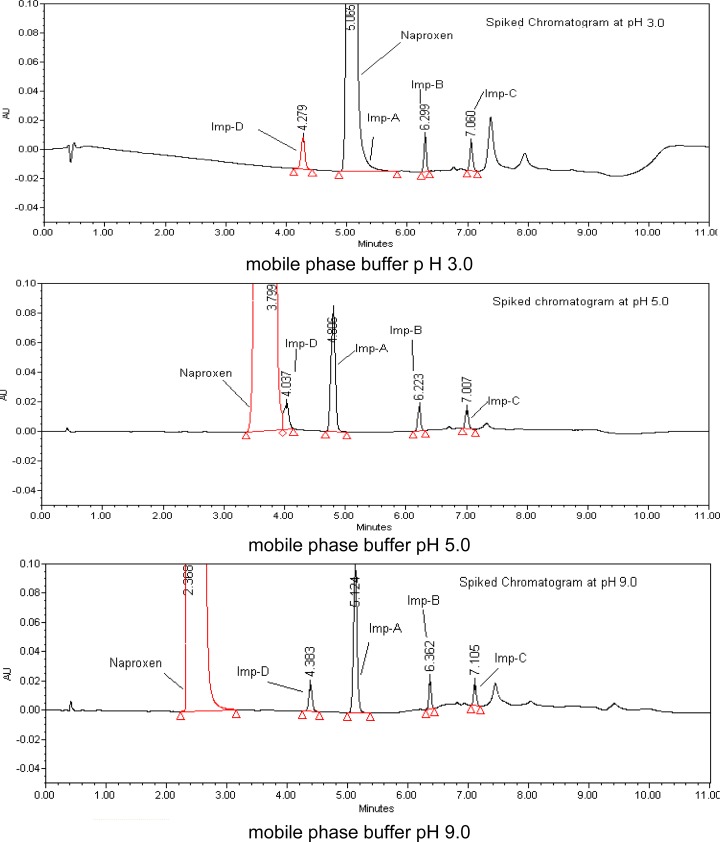
Typical chromatograms of Naproxen from method development trails

The main target of the chromatographic method is to achieve the separation of impurities and the main component NAP. The maximum absorption value for NAP and its degradation products is 260 nm (Fig. 2), hence its selection as the detection wavelength for the analysis. A blended solution containing 2500 μg/ml of NAP and 2.5 μg/ml of each impurity dissolved in diluent was used for the method’s development. Initial experiments were performed with glacial acetic acid in water as a buffer, so that Naproxen and its impurities were retained on the column. The retention behavior of Naproxen and its impurities were dependent on the content of the organic modifier in the mobile phase. Initially, a mobile phase composed of potassium dihydrogen orthophosphate solution (20mM) and methanol (70:30 v/v) flowing at a rate of 0.3 mL/min over Zorbax XDB (C-18, 50-mm×2.1-mm, 1.8 μm particles) columns was employed for NAP. The drug and its eluted impurities with highly asymmetric peak shapes (USP tailing was more than 2.0) along with Imp-C were highly retained. The variation in the organic modifier proportions in the mobile phase also did not produced a pure, symmetrical peak from the Inertsil and Zorbax columns. Better peak shape was observed in the Acquit BEH C18 column when compared to other columns with a similar stationary phase. After the addition of triethylamine to the buffer which affected the peak shape of the drug, the use of acetonitrile in the mobile phase affected the retention time of Imp-C, which resulted in peak tailing proportional to the organic modifier in the mobile phase B. The addition of methanol as the organic modifier along with acetonitrile resulted in optimum peak shape. To check the retention behavior of Naproxen in different pH conditions, the mobile pH adjusted to 3.0, 5.0, 7.0, and 9.0and the Naproxen impurity mix was injected before finalizing the mobile pH. By decreasing the pH value from 9.0 to 3.0, Naproxen retention time increased from 2.4 to 5.0 and imp-D merged with Naproxen at pH 5.0; there was no pH impact on retentions of Naproxen-related compounds (Fig. 3). From pH 7.0 to 9.0 there was not much difference in retention times of Naproxen and its related compounds, so pH 7.0 was finalized as the mobile phase buffer pH. The eluted analyte retention time was approximately 2.4 min. Interference with the excipients (placebo) was also checked, and no interference was observed between the impurity peaks and the NAP peak. Several preliminary chromatographic runs were performed to investigate the suitability of the drug content estimation and cost because of the increasing importance of rapid economic analysis in pharmaceutical analysis to increase the throughput. The system suitability parameters were evaluated for NAP and its four impurities. The USP tailing factor for all four impurities and NAP was found to be less than 1.4.

### Method validation

#### Results from forced degradation studies

The stress conditions used for the degradation study of Naproxen included light (conducted as stipulated in ICH Q1B), heat (105 °C for 5 hrs), acid hydrolysis (1 N HCl at 60 °C for 2hrs), basic hydrolysis (1 N NaOH at 60 °C for 6 hrs), aqueous hydrolysis (60 °C for 6 hrs), and oxidation (6 % H_2_O_2_ at 40 °C for 2hrs). For studies of the effects of light, the study period was 10 days. Degradation was observed when Naproxen was subjected to acid and base degradation conditions, but degradation was not observed when subjected to peroxide, water, humidity, heat, and photolytic conditions. Naproxen converted 17% to imp-4 which formed during acid degradation. The mass balance (% assay + % sum of all compounds + % sum of all degradants) results were calculated for all of the stressed samples and were found to be more than 95 % (Table 1). The purity and assay of NAP was unaffected by the presence of its impurities and degradation products, which confirms the stability-indicating power of the developed method (Fig. 2).

#### Precision

The repeatability of the test method for the related substances was checked by a sixfold analysis of 2500 μg/ml of Naproxen spiked with 2.5 μg/ml of each of the four impurities. The RSD (%) of peak area was calculated for each impurity. Inter-and intra-day variation and analyst variation were studied to determine the intermediate precision of the proposed method. The %RSD of NAP during the assay method precision was 0.4% and 0.2 % for intermediate precision. The % RSD of the area for each impurity was calculated for both precision and intermediate precision and was found to be within 2 %. These results confirmed the precision and ruggedness of the method (Table 2).

#### Limits of Detection and Limit of Quantification (LOD & LOQ)

Limit of detection (LOD) is the lowest amount of analyte which can be detected, but not necessarily quantitated as an exact value; limit of quantification is the lowest amount of analyte which can be determined with suitable precision and accuracy. Both LOD and LOQ values are determined using the signal-to-noise ratio method. The analyte concentration at which the S/N value is around 3 is considered as the LOD and 10 is considered as the LOQ. The LOD and LOQ values of Naproxen and related substances are given in Table 2.

#### Accuracy

**Fig. 4.**
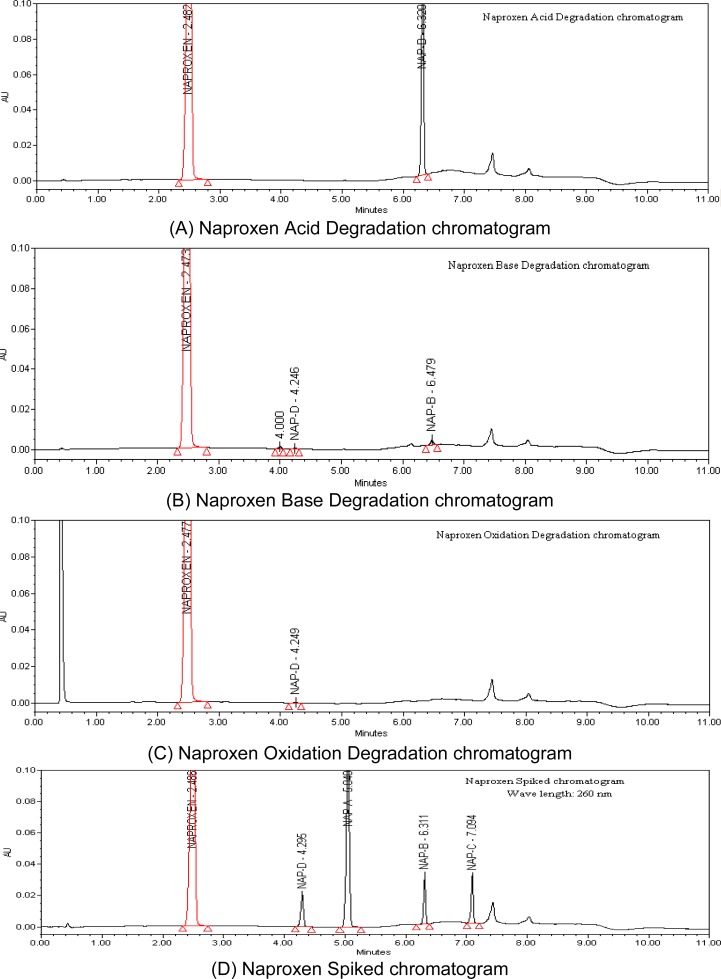
Degradation and Impurity Spiked Chromatograms of Naproxen from A to D

The accuracy of an analytical procedure expresses the closeness of agreement between the reference value and the value found. Impurities’ recovery was determined in triplicate from the LOQ to 0.15 % of the Naproxen test concentration. The recovery of Naproxen from pharmaceutical dosage forms ranged from 98.6 to 101.4 %. The recovery of Naproxen and impurities from pharmaceutical dosage forms ranged from 97.8 to 102.5 %. A UPLC chromatogram obtained from a sample of Naproxen (0.1%) spiked with its impurities is shown in Fig. 4.

#### Linearity

The linearity of an analytical procedure is its ability to obtain test results which are directly proportional to the concentration of the analyte in the sample. The linearity of test method was established from the LOQ to 200 % of the test concentration for Naproxen and its related substances. The correlation coefficient obtained for Naproxen and its related substances was >0.998 (Table 2). The linearity calibration plot for the assay method was obtained over the calibration ranges tested, and the correlation coefficient obtained was greater than 0.999. The result showed that an excellent correlation existed between the peak area and concentration of the analyte. This confirmed the linear relationship between peak areas and concentrations. The results indicate very good linearity.

#### Robustness

The robustness of an analytical procedure is a measure of its capacity to remain unaffected by small, but deliberate variations in method parameters and provides an indication of its reliability during normal usage. In all the varied chromatographic conditions (flow rate, column temperature, and pH of the mobile phase), all analyte peaks were adequately separated and there was no change in the elution order of Naproxen and its impurities.

#### Stability in solution and in the mobile phase

The RSD (%) values for the NAP assay during the solution stability and mobile phase stability experiments were within 1.5 %. No significant changes in the amounts of the four impurities were observed during the solution stability (on the benchtop) and mobile phase experiments when performed by the related substances method. The results from the solution stability and mobile phase stability experiments confirmed that the standard solutions and solutions in the mobile phase were stable for up to 24 h during the assay and determination of related substances.

## Conclusion

The rapid gradient RP-UPLC method was developed and validated for the determination of related substances in pharmaceutical dosage forms of Naproxen. The developed method was found to be precise, accurate, linear, robust, and specific. The total runtime of the method was 11 minutes within which all the impurities related to Naproxen were well-separated. This novel UPLC method reduces the analysis time and cost, and increases the effective utilization of the instrument and column. This method also exhibited excellent performance in terms of sensitivity and speed. The method is stability-indicating and can be used for the routine analysis of production samples and to check the stability of Naproxen samples.

## Figures and Tables

**Tab. 1.** Naproxen Forced degradation data in all conditions

**Parameter**	**Naproxen**	**Imp-A**	**Imp-B**	**Imp-C**	**Imp-D**	**% net Degradation**	**Mass Balance**
Base	99.42	Nil	0.36	Nil	0.08	0.58	99.4
Acid	83	Nil	16.9	Nil	Nil	17	98.8
Heat	99.9	Nil	Nil	Nil	0.02	0.1	99.9
Water	99.2	Nil	Nil	Nil	0.07	0.8	99
Peroxide	99.9	Nil	Nil	Nil	0.08	0.1	99.9
Sunlight	99.9	Nil	Nil	Nil	0.02	0.1	99.5
Humidity	99.9	Nil	Nil	Nil	0.01	0.1	99.5

**Tab. 2.** LOD, LOQ, regression data, Precision:

**Parameter**	**Naproxen**	**Imp-A**	**Imp-B**	**Imp-C**	**Imp-D**
LOD (%)	0.008	0.005	0.01	0.02	0.004
LOQ (%)	0.02	0.01	0.03	0.04	0.01
Regression equation					
Slope (b)	62021.1	62021.1	51543.1	46869.9	110416.9
Intercept (a)	260.8	260.8	244.6	594.6	618.9
Correlation Coefficient	0.999	0.999	0.999	0.999	0.999
Precision (%RSD)	0.5	0.6	1.2	0.5	1.7
Intermediate Precision (%RSD)	0.6	0.7	0.8	0.9	1.2
